# Construction and internal temporal validation of a LASSO regression-based risk assessment model for non-suicidal self-injury addiction-like features in adolescents and young adults with depression

**DOI:** 10.3389/fpsyg.2026.1895600

**Published:** 2026-07-22

**Authors:** Houqin Huang, Fengmei Lu, Shuiqin Cao, Chuan Jiang, Jianyan Peng, Yuhao Tong, Pengyuan Wang, Yi Liu, Zixiang Ye

**Affiliations:** 1 MOE Key Laboratory for Neuroinformation, Department of Nursing, The Clinical Hospital of Chengdu Brain Science Institute, University of Electronic Science and Technology of China, Chengdu, China; 2 Affiliated Hospital of Chengdu University, Chengdu, China; 3 Acute Psychiatry, Chengdu Jinxin Mental Hospital, Chengdu, China

**Keywords:** addiction-like features, adolescents, depression, LASSO regression, nomogram, non-suicidal self-injury, risk assessment model, temporal validation

## Abstract

**Background:**

Non-suicidal self-injury (NSSI) is common among adolescents and young adults with depression and may present addiction-like features, increasing clinical complexity and suicide risk. However, clinically applicable tools for estimating the likelihood of NSSI addiction-like features remain limited. This study aimed to develop and internally temporally validate a LASSO-based cross-sectional risk assessment model for NSSI addiction-like features among patients with depression.

**Methods:**

A total of 511 adolescents and young adults with depression were included. All candidate predictors and the outcome were assessed at the same clinical evaluation; therefore, the model was interpreted as an association-based risk stratification tool rather than as evidence of causal or prospective prediction. Participants recruited during the first 3 months were assigned to the training cohort, and those recruited during the final month were assigned to the internal temporal validation cohort. LASSO regression was used for variable selection, and the selected variables were entered into multivariable logistic regression to construct the model and nomogram. Model performance was evaluated using ROC curves, calibration curves, calibration intercept and slope, Brier score, bootstrap validation, Hosmer-Lemeshow goodness-of-fit test, and decision curve analysis.

**Results:**

Among the 511 participants, 55.58% met the operational criterion for NSSI addiction-like features. LASSO regression identified age at first self-injury, gender, NSSI severity, borderline personality features, family functioning, and childhood trauma as the main associated variables. Older age at first self-injury, male gender, and better family functioning were associated with lower odds of NSSI addiction-like features, whereas greater NSSI severity, more prominent borderline personality features, and childhood trauma were associated with higher odds. The model yielded AUCs of 0.9366 in the training cohort and 0.9086 in the internal temporal validation cohort. The optimism-corrected AUC was 0.9310, and the more conservative bootstrap-corrected AUC was 0.9181. Brier scores were 0.1000 and 0.1220, respectively. In the validation cohort, the calibration intercept and slope were −0.1283 and 0.8895.

**Conclusion:**

NSSI addiction-like features were common in this clinical sample. The model showed promising discrimination, calibration, and potential clinical utility for cross-sectional risk stratification, but requires independent validation and prospective testing before routine clinical implementation.

## 1 Introduction

According to China’s seventh national census, the population aged 10–29 years was approximately 320 million, accounting for about 23% of the national population (Liu et al., 2024). The latest Report on the Development of National Mental Health in China (2019–2020) showed that the detection rate of depression among Chinese adolescents was 24.6%. Adolescent depression refers to depression occurring during adolescence, commonly defined as 12–18 years of age. Previous studies have reported that the prevalence of adolescent depression ranges from 1.8 to 7.8%, and that the 12-month prevalence in middle to late adolescence is similar to that in adults, ranging from 4.0 to 7.5% (Shorey et al., 2022; Ye et al., 2023).

Non-suicidal self-injury (NSSI) refers to the intentional and direct destruction of one’s own body tissue without suicidal intent, including socially unacceptable behaviors such as cutting, burning, and hitting oneself. Among hospitalized adolescents, approximately 30–45% engage in NSSI (Juli et al., 2024). In community samples, the prevalence of NSSI among adolescents ranges from 9.9 to 35.6% (Xie et al., 2024; Poudel et al., 2022). NSSI not only increases the risk of suicide but also causes physical harm, including scarring and wound infection (He et al., 2023). Beyond psychosocial factors, neuroimaging research in adolescents with bipolar disorder has identified resting-state functional connectivity patterns associated with risk and resilience for self-harm (Dimick et al., 2023). Some adolescents with depression report addiction-like experiences related to NSSI, such as recurrent urges, loss of control, repeated engagement despite negative consequences, and temporary relief or pleasure following self-injury (Guo et al., 2023; Davis and Lewis, 2019). The addiction framework is theoretically supported by negative reinforcement models, in which self-injury rapidly reduces aversive affect and thereby strengthens the behavior over time, and by clinical descriptions of craving, tolerance-like escalation, and withdrawal-like tension before self-injury (Tang et al., 2020; Himelein-Wachowiak et al., 2022). Nevertheless, this construct is not a formal independent diagnosis in DSM-5 or ICD-11, and the addiction framework remains debated. In the present study, the term “NSSI addiction-like features” is used to emphasize that the outcome reflects clinically relevant addictive characteristics measured by a validated self-report subscale rather than a formal diagnostic category. The addiction subscale of the Ottawa Self-Injury Inventory (OSI) was selected because it was developed based on substance-dependence criteria and has been used in prior studies to operationalize addictive features of NSSI (Guérin-Marion et al., 2018; Luo et al., 2024).

Recent research has increasingly applied nomograms and machine-learning approaches to identify adolescents at risk for NSSI. For example, Zhong et al. (2024) developed a machine-learning model for NSSI risk among adolescents in western China, Sun et al. (2024) constructed XGBoost and random-forest models for NSSI among Chinese adolescents with major depressive disorder, and Li et al. (2025) developed a longitudinal nomogram for NSSI in youth with depression. Other nomogram studies have also focused on NSSI behaviors among adolescents with depression or specific student populations (Gao et al., 2025; Zhao et al., 2025). These studies provide important evidence for early identification of NSSI behavior, but most have treated NSSI as a binary behavioral outcome rather than focusing on addiction-like features of NSSI. Therefore, the incremental contribution of the present study is its focus on addiction-like features of NSSI among adolescents and young adults with depression, its use of clinically accessible psychological and family-related variables, and its explicit evaluation of calibration, optimism-corrected performance, multicollinearity, and decision-curve net benefit.

LASSO regression uses the sum of the absolute values of regression coefficients as a penalty term, shrinking the coefficients of independent variables that are weakly associated with the dependent variable to zero. This approach helps identify variables most strongly associated with the outcome, reduces model complexity, and improves model parsimony (Kang et al., 2021). Therefore, this study used LASSO regression to screen variables associated with NSSI addiction-like features in adolescents and young adults with depression, construct an association-based risk assessment model, and develop a nomogram. The model is intended to support early clinical risk stratification and hypothesis generation, but it should not be interpreted as establishing causality or prospective prediction without longitudinal validation.

## 2 Methods

### 2.1 Study design and participants

A total of 511 patients with depression who visited the psychiatric outpatient department of a tertiary hospital in Chengdu between December 1, 2024 and March 30, 2025 were selected as study participants. Data from the first 3 months were used as the training cohort, and data from the final month were used as an internal temporal validation cohort. The inclusion criteria were as follows: (1) age 13–20 years, with no restriction on gender (Lu et al., 2024); although the World Health Organization generally defines adolescence as 10–19 years, 20-year-old patients were included because the participating clinical service commonly manages late-adolescent/transition-age youth in the same outpatient pathway, and because developmental, educational, and family-context characteristics may remain similar in this transition period; (2) meeting the diagnostic criteria for depression in the 10th Revision of the International Classification of Diseases and the diagnostic criteria for NSSI in the fifth edition of the Diagnostic and Statistical Manual of Mental Disorders; both first-episode and recurrent depression were eligible, with or without psychotic symptoms; (3) primary school education or above and ability to understand the scale content; and (4) both the patient and guardian agreed to participate and signed informed consent. The exclusion criteria were as follows: (1) previous diagnosis of schizophrenia, alcohol dependence, organic mental disorder, or other psychiatric disorders; (2) serious physical illness that the researchers considered unsuitable for participation; and (3) religious or culturally customary self-injury. The criteria for determining NSSI addiction-like features were as follows: the addiction subscale of the OSI consists of seven items developed with reference to DSM-IV substance dependence criteria. A score greater than 2 on more than three items indicates addictive features. Participants were divided into non-addiction-like and addiction-like groups according to whether they met this operational criterion (Luo et al., 2024). On the basis of previous literature (Shao et al., 2021; Liu et al., 2023; Yang et al., 2025; Lee et al., 2021; Zhu et al., 2020; You et al., 2024; Liang et al., 2023), 20 related variables were included. The events per variable (EPV) method was used to calculate sample size, requiring the number of outcome events to be no less than 10–20 times the number of included independent variables. This study adopted an EPV of 10 and allowed for a 10% data loss rate; the final calculated sample size for the modeling set was 220 cases (20 × 10/0.9). Ultimately, 341 cases were included in the training cohort. This study was approved by the Ethics Committee of the Fourth People’s Hospital of Chengdu (Ethics Approval No. 2025-Ethics Review-51-01).

### 2.2 Data collection

Data were collected from all participants using a self-designed general information questionnaire and validated Chinese versions of standardized instruments. Depressive symptoms were assessed using the 17-item Hamilton Depression Rating Scale (HAMD-17; range 0–52), and anxiety symptoms were assessed using the Hamilton Anxiety Rating Scale (HAMA-14; range 0–56), with higher scores indicating more severe symptoms. NSSI-related characteristics were assessed using the Chinese version of the Ottawa Self-Injury Inventory (OSI). The OSI addiction subscale consists of seven items developed with reference to DSM-IV substance-dependence criteria; participants who scored greater than 2 on more than three items were classified as having addiction-like features of NSSI. NSSI severity was calculated from OSI items related to NSSI frequency, methods, and difficulty controlling the behavior, with higher scores indicating more severe NSSI. Borderline personality features were assessed using the Chinese version of the Personality Diagnostic Questionnaire-4 + borderline personality disorder subscale (PDQ-4 + BPD subscale). In this study, borderline personality features were analyzed as a continuous trait score rather than as a categorical clinical diagnosis, with higher scores indicating more prominent borderline personality features. Family functioning was assessed using the Chinese version of the Family APGAR Index, which contains five items assessing adaptation, partnership, growth, affection, and resolve; total scores range from 0 to 10, with higher scores indicating better family functioning. Social support was assessed using the Chinese version of the Social Support Rating Scale (SSRS), with higher scores indicating greater social support. Rumination, childhood trauma, adverse life events, loneliness, peer relationships, school attitude, academic pressure, interpersonal relationships, and school bullying were assessed using validated Chinese instruments or structured questionnaires. For all scales, higher scores represented higher levels of the corresponding construct unless otherwise specified. The specific scale versions, scoring ranges, score interpretations, and Cronbach’s alpha coefficients in the present sample are provided in Supplementary Table S2.

To further improve measurement transparency, the revised Supplementary Table S2 lists the full name of each instrument, the principal original or source reference used to support the measure, scoring range, score direction, and Cronbach’s alpha coefficient in the present sample.

### 2.3 Statistical methods

Statistical analyses were performed using SPSS 21.0 and R 4.1.1. Cases with missing values of 20% or more were excluded, and variables with missing values of less than 20% were handled using multiple imputation by chained equations. The number and percentage of missing values were examined for each candidate predictor and the outcome variable. All candidate predictors and the outcome variable were included in the multiple-imputation model to preserve associations among variables. Missing-data patterns are reported in Supplementary Table S1. Count data were described as frequencies, percentages, or rates, and between-group comparisons were performed using the chi-square test. Normally distributed continuous variables were described as means and standard deviations, and between-group comparisons were performed using the independent-samples t test. Non-normally distributed continuous variables were described as medians and interquartile ranges, and between-group comparisons were performed using the Mann–Whitney U test. LASSO regression was used for variable reduction because it can retain key predictors while addressing multicollinearity and improving model parsimony (Wang Q. et al., 2022). LASSO regression analysis was conducted using the glmnet package in R 4.1.1, and 10-fold cross-validation was used to determine the regularization parameter lambda. Variables selected by LASSO were then entered simultaneously into a multivariable logistic regression model; no additional stepwise selection was performed in the revised analysis to avoid instability and selection bias. A risk assessment model was constructed and a nomogram was drawn using the rms package. Discrimination was assessed using the AUC with 95% confidence intervals; bootstrap resampling with 1,000 repetitions was used to estimate optimism-corrected performance. Calibration was evaluated using calibration curves, calibration intercept, calibration slope, Brier score, and the Hosmer-Lemeshow goodness-of-fit test. Multicollinearity in the final logistic model was assessed using variance inflation factors (VIFs), with VIF < 5 considered acceptable. Decision curve analysis was used to assess clinical net benefit across threshold probabilities. A two-sided *p* value <0.05 was considered statistically significant.

**Figure 1:**
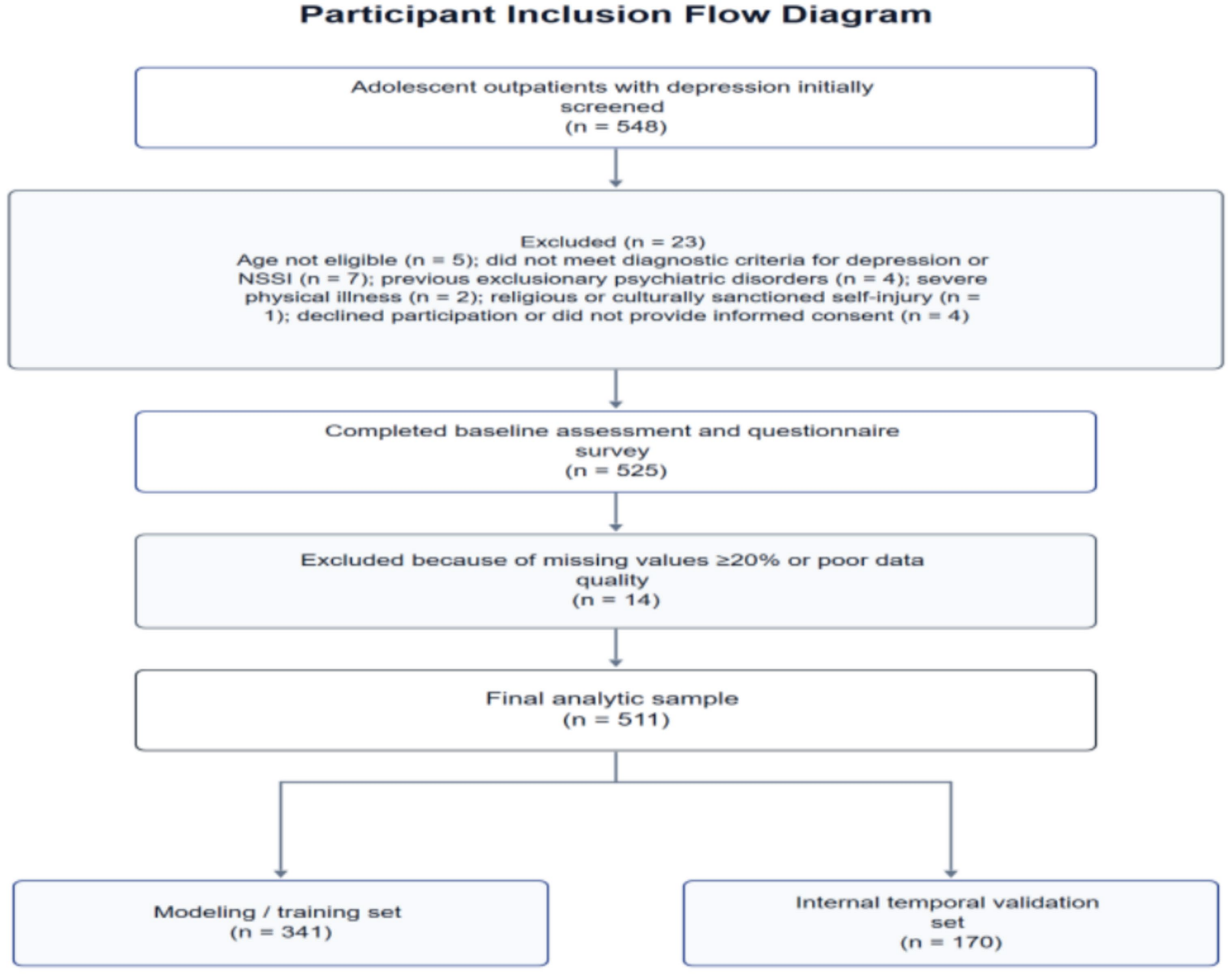
Participant inclusion flow diagram.

To minimize information leakage, the temporal cohort assignment was defined before imputation and model development. Multiple imputation was performed within the training cohort and internal temporal validation cohort rather than using outcome or predictor distributions pooled across both cohorts. LASSO variable selection and estimation of the final logistic regression coefficients were conducted exclusively in the training cohort; the resulting model equation was then applied unchanged to the internal temporal validation cohort to evaluate discrimination, calibration, and clinical net benefit. The participant inclusion flow diagram is presented in Figure 1.

## 3 Results

### 3.1 General characteristics of adolescents and young adults with depression and incidence of NSSI addiction-like features

A total of 511 participants were included, and 284 (55.58%) met the operational criterion for NSSI addiction-like features. The training cohort comprised 341 cases, including 194 cases with NSSI addiction-like features (56.89%); the internal temporal validation cohort comprised 170 cases, including 90 cases with NSSI addiction-like features (52.94%). Significant differences were observed between participants with and without NSSI addiction-like features in age at first self-injury, monthly income, parental education level, depression score, NSSI severity, borderline personality feature score, family functioning score, academic pressure, and childhood trauma (all *p* < 0.05), as shown in Table 1. The difference in sexual orientation should be interpreted with caution because only five participants reported a non-heterosexual orientation, limiting statistical reliability. Of the 548 patients initially screened, 23 were excluded before enrollment because they did not meet eligibility criteria or did not provide consent, and 14 were further excluded because of missing values ≥20% or poor data quality. Finally, 511 participants were included in the analysis (Figure 1). Missing values for candidate predictors ranged from 0 to 7 cases (0.00–1.37%); no variable had a missing proportion of 20% or higher (Supplementary Table S1).

**Table 1:** Univariate analysis of factors associated with NSSI Addiction-Like features in adolescents and young adults with depression.

Item	Category	Non-addiction group (*n* = 227)	Addiction group (*n* = 284)	Statistic	*P*
Age at first self-injury (years)		14.49 ± 1.40	13.59 ± 1.88	t = 6.201	<0.001
Gender [*n* (%)]				χ^2^ = 54.619	<0.001
	Female	74 (32.6)	186 (65.5)		
	Male	153 (67.4)	98 (34.5)		
Sexual orientation [*n* (%)]				χ^2^ = 0.040	0.841
	Heterosexual	225 (99.1)	281 (98.9)		
	Non-heterosexual	2 (0.9)	3 (1.1)		
Monthly income [*n* (%)]				χ^2^ = 11.926	0.003
	<3,000	9 (4.0)	25 (8.8)		
	>5,000	116 (51.1)	169 (59.5)		
	Other/unknown	102 (44.9)	90 (31.7)		
Parental education level [*n* (%)]				χ^2^ = 9.151	0.010
	Primary school or below	66 (29.1)	107 (37.7)		
	Junior/senior high school	132 (58.1)	127 (44.7)		
	College or above	29 (12.8)	50 (17.6)		
Depression score		22.91 ± 3.88	23.76 ± 5.16	t = −2.113	0.035
Anxiety score		22.20 ± 3.84	22.33 ± 4.16	t = −0.362	0.717
NSSI severity score		6.01 ± 2.39	9.06 ± 2.11	t = −15.072	<0.001
Adverse life events score		52.16 ± 10.01	50.79 ± 11.22	t = 1.453	0.147
Borderline personality disorder score		61.49 ± 12.20	69.42 ± 16.33	t = −6.283	<0.001
Rumination score		60.99 ± 6.56	60.39 ± 7.15	t = 0.980	0.328
Family functioning score		5.46 ± 1.64	4.02 ± 1.73	t = 9.586	<0.001
Loneliness score		5.21 ± 0.80	5.33 ± 0.83	t = −1.662	0.097
Peer relationship score		2.72 ± 0.62	2.71 ± 0.56	t = 0.061	0.951
Attitude toward school score		84.29 ± 4.73	84.00 ± 4.94	t = 0.666	0.506
School bullying [*n* (%)]				χ^2^ = 0.466	0.495
	No	114 (50.2)	134 (47.2)		
	Yes	113 (49.8)	150 (52.8)		
Academic pressure score		15.38 ± 2.08	14.83 ± 2.10	t = 2.946	0.003
Interpersonal relationship score		14.00 ± 2.55	13.88 ± 2.27	t = 0.558	0.577
Social support score		51.44 ± 9.69	51.45 ± 7.31	t = −0.013	0.990
Childhood trauma score		47.22 ± 7.00	50.63 ± 6.40	t = −5.688	<0.001

### 3.2 Variable selection based on LASSO regression

**Figure 2:**
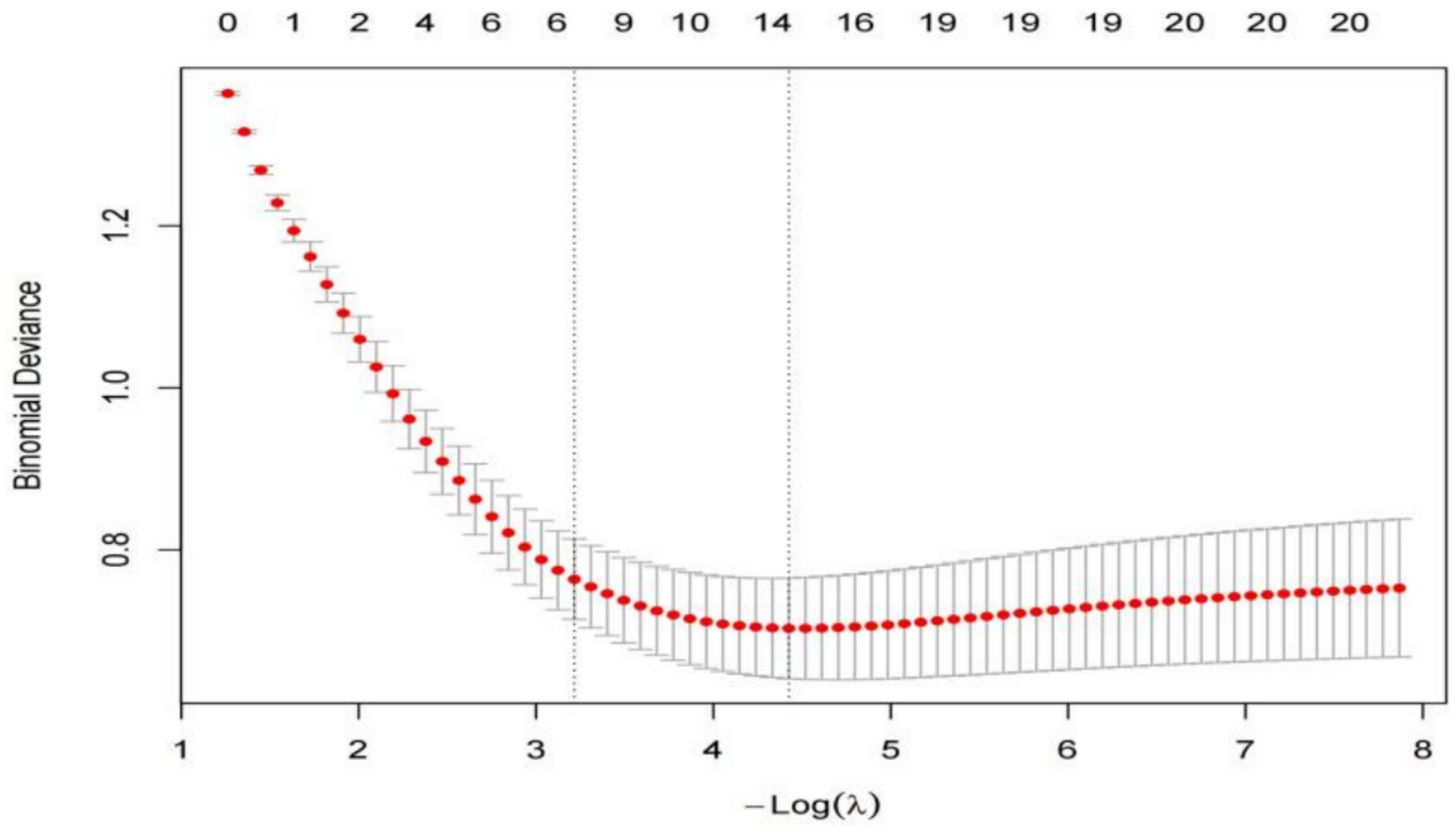
Ten-fold cross-validation plot.

**Figure 3:**
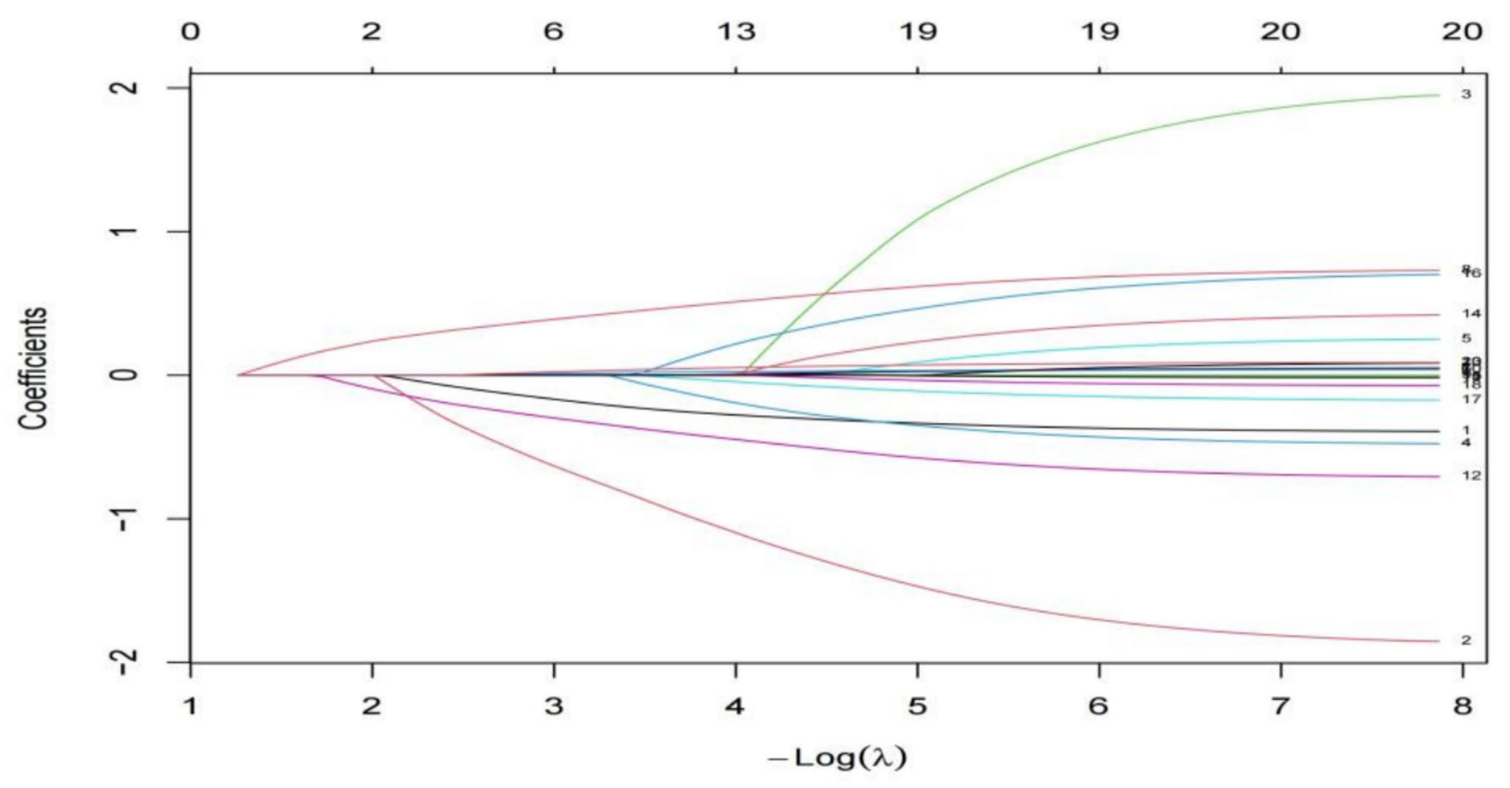
LASSO regression coefficient path plot.

In the training cohort, all 20 candidate variables were included in the LASSO regression. Ten-fold cross-validation was used to select the optimal lambda value. When log (lambda) corresponded to lambda.1se = 0.0401145, six variables - age at first self-injury, gender, NSSI severity, borderline personality features, family functioning, and childhood trauma - were identified as variables associated with NSSI addiction-like features. The changes in variables with lambda in the LASSO model are shown in Figures 2, 3.

### 3.3 Multivariate analysis of factors influencing NSSI addiction-like features in adolescents and young adults with depression

Whether participants with depression met the operational criterion for NSSI addiction-like features was used as the dependent variable, and the six variables selected by LASSO were entered simultaneously as independent variables in multivariable logistic regression. Variable assignments are shown in Table 2. To improve interpretability, gender was coded as female = 0 and male = 1 in the revised explanation, so the odds ratio represents the odds for males compared with females. The results showed that older age at first self-injury, male gender, and better family functioning were associated with lower odds of NSSI addiction-like features, whereas greater NSSI severity, more prominent borderline personality features, and childhood trauma were associated with higher odds, as shown in Table 3. Multicollinearity diagnostics showed that all VIF values were close to 1 and below the conventional threshold of 5 (age at first self-injury, 1.087; gender, 1.065; NSSI severity, 1.167; borderline personality features, 1.035; family functioning, 1.071; childhood trauma, 1.029), indicating no apparent multicollinearity among the final predictors. Detailed VIF values are provided in Supplementary Table S3.

**Table 2:** Variable assignments.

Variable	Assignment method
Age at first self-injury	Entered as original value
Gender	Female = 0, male = 1
Sexual orientation	Non-heterosexual = 1, heterosexual = 2
Monthly income	<3,000 = 1, 3,001–5,000 = 2, >5,000 = 3
Parental education level	Primary school or below = 1, junior/senior high school = 2, college or above = 3
Depression	Entered as original value
Anxiety	Entered as original value
NSSI severity	Entered as original value
Adverse life events	Entered as original value
Borderline personality disorder	Entered as original value
Family functioning	Entered as original value; higher scores indicate better family functioning
Rumination	Entered as original value

**Table 3:** LASSO-logistic regression analysis of factors associated with NSSI addiction-like features in adolescents and young adults with depression.

Variable	Regression coefficient	Standard error	Wald χ^2^	*P*	OR
Intercept	−0.336	2.307	0.021	0.884	-
Age at first self-injury	−0.427	0.110	15.144	<0.001	0.652
Gender	−1.518	0.355	18.242	<0.001	0.219
NSSI severity	0.640	0.091	49.786	<0.001	1.896
Borderline personality disorder	0.041	0.012	11.104	0.001	1.042
Family functioning	−0.603	0.108	31.225	<0.001	0.547
Childhood trauma	0.089	0.027	11.067	0.001	1.093

### 3.4 Nomogram of the risk assessment model for NSSI addiction-like features in adolescents and young adults with depression

**Figure 4:**
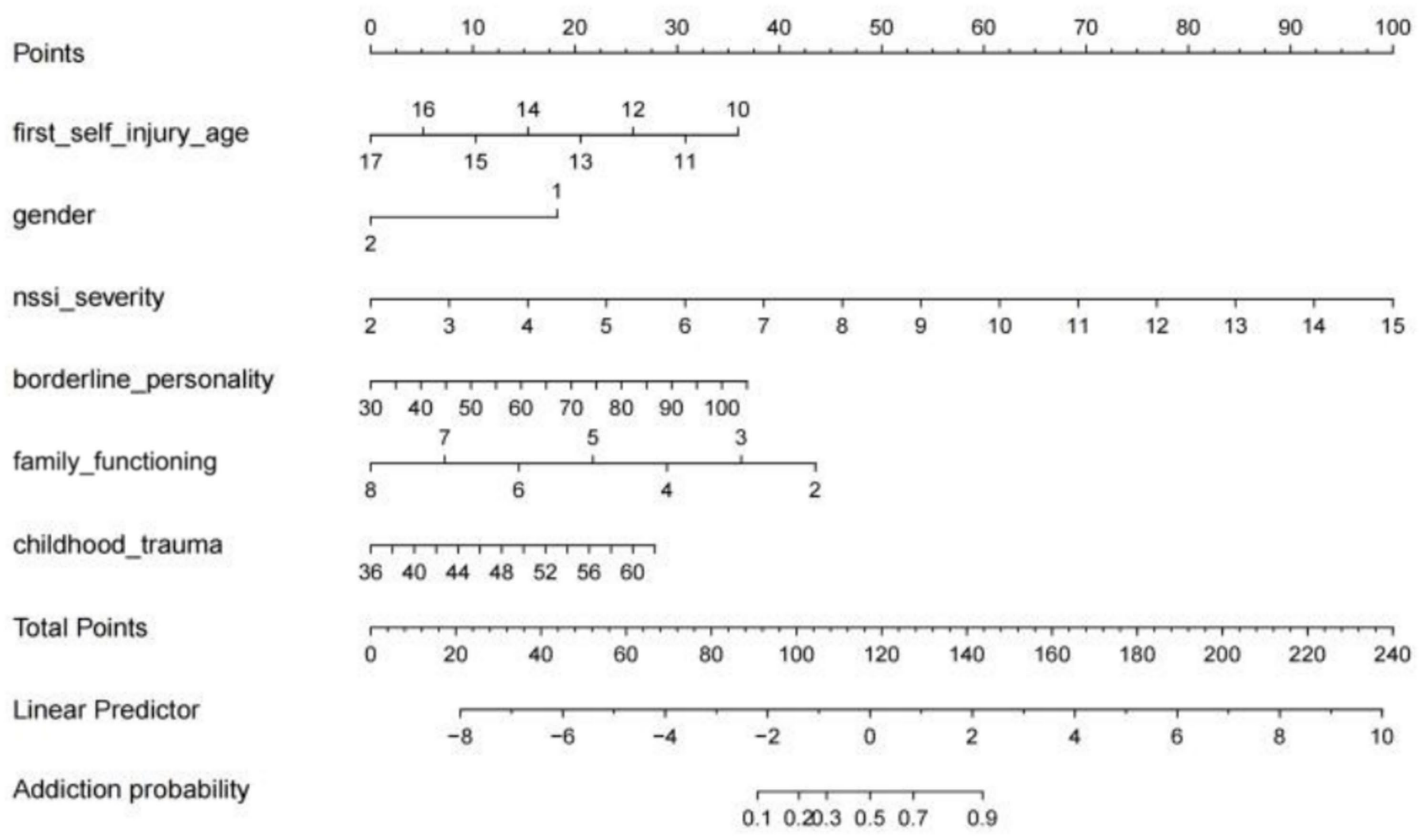
Nomogram for estimating the risk of NSSI addiction-like features in adolescents and young adults with depression using the LASSO-logistic regression model.

According to the LASSO-logistic regression model, the final risk assessment model for NSSI addiction-like features was as follows: logit (P) = −0.336 - 0.427 x age at first self-injury −1.518 x gender (male vs. female) + 0.640 x NSSI severity score + 0.041 x borderline personality feature score - 0.603 x family functioning score + 0.089 x childhood trauma score. A nomogram was developed based on this model, as shown in Figure 4. In practical use, clinicians can locate each patient’s value on the corresponding nomogram axis, sum the points across predictors, and convert the total points to the estimated probability of NSSI addiction-like features. For example, a female patient with earlier onset of self-injury, high NSSI severity, high borderline personality feature score, poor family functioning, and high childhood trauma score would obtain a higher total score and would be classified as needing closer risk assessment and targeted intervention (Table 4).

**Table 4:** Apparent, bootstrap-corrected, and temporal validation performance of the risk assessment model.

Metric	Training cohort apparent value	Bootstrap-corrected value	Internal temporal validation cohort
AUC	0.9366	0.9310 (0.9181 when the full modeling procedure including LASSO selection was bootstrapped)	0.9086
Brier score	0.1000	0.1061	0.1220
Calibration intercept	0.0000	0.0034	−0.1283
Calibration slope	1.0000	0.9442	0.8895

### 3.5 Validation of the risk assessment model for NSSI addiction-like features in adolescents and young adults with depression

**Figure 5:**
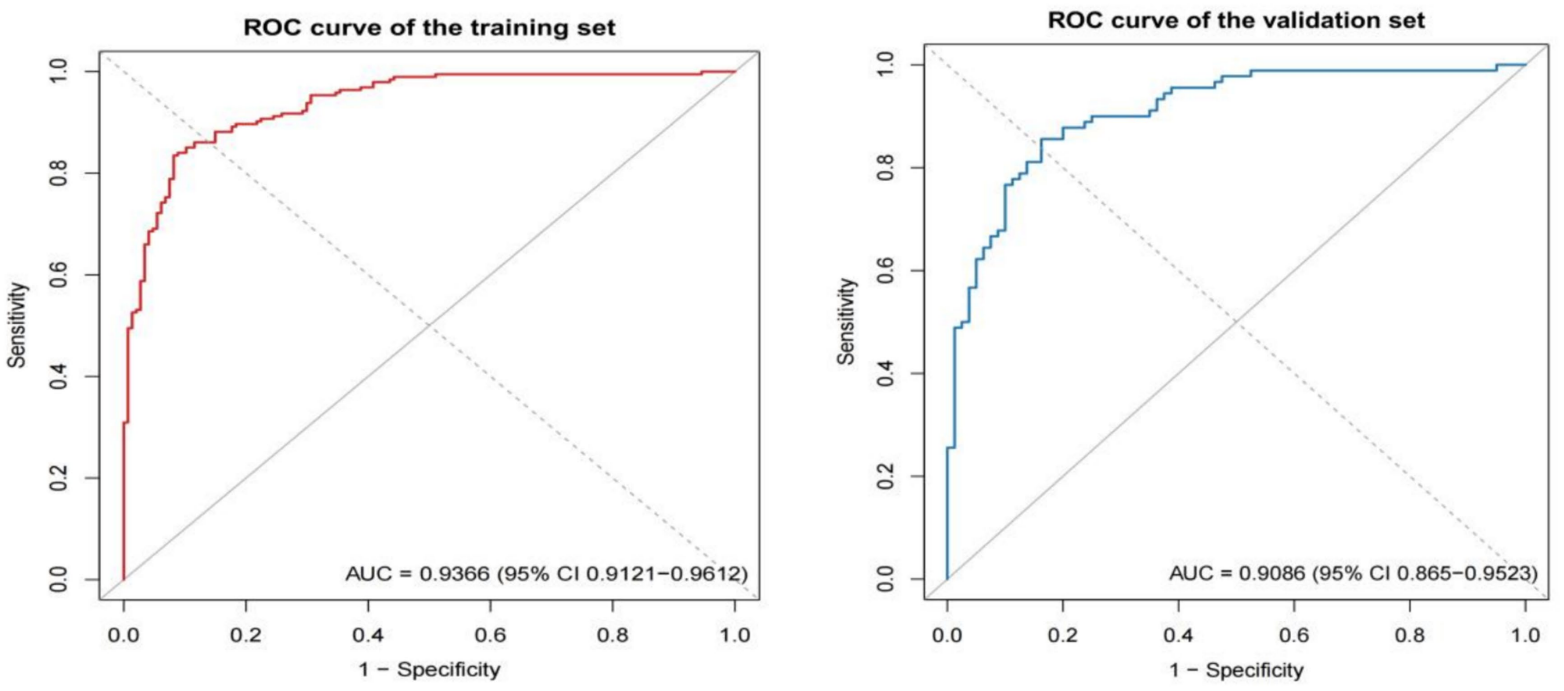
ROC curves of the risk assessment model in the training and internal temporal validation cohorts.

In the training cohort, the model had an apparent AUC of 0.9366 (95% CI: 0.912–0.961), with a sensitivity of 83.51% and a specificity of 91.8%, indicating good discriminatory ability (Figure 5). Bootstrap internal validation with 1,000 repetitions yielded an optimism-corrected AUC of 0.9310 for the final model, corresponding to an estimated optimism of 0.0056. When the entire modeling procedure, including LASSO variable selection, was repeated during bootstrap resampling, the more conservative optimism-corrected AUC was 0.9181. Calibration analysis showed an apparent calibration intercept of 0.0000, apparent calibration slope of 1.0000, and Brier score of 0.1000 in the training cohort. After bootstrap correction, the calibration intercept was 0.0034, the calibration slope was 0.9442, and the Brier score was 0.1061. The mean absolute error of the calibration curve was 0.039. The Hosmer-Lemeshow goodness-of-fit test yielded χ^2^ = 6.797 and *p* = 0.559, suggesting no strong evidence of lack of fit.

The temporal validation analysis used the exact model derived from the training cohort, without reselecting variables or refitting coefficients in the validation cohort.

**Figure 6:**
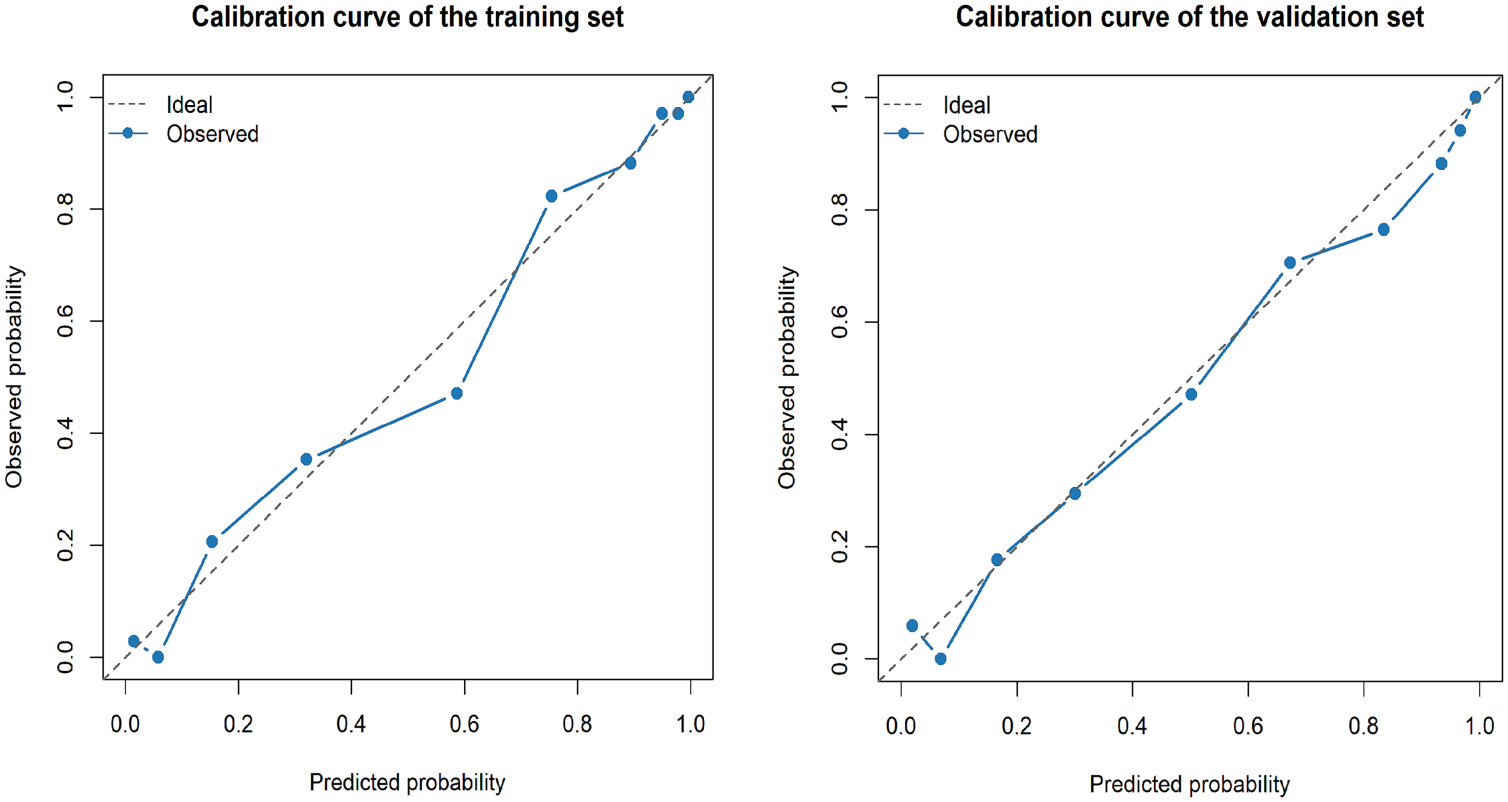
Calibration curves.

**Figure 7:**
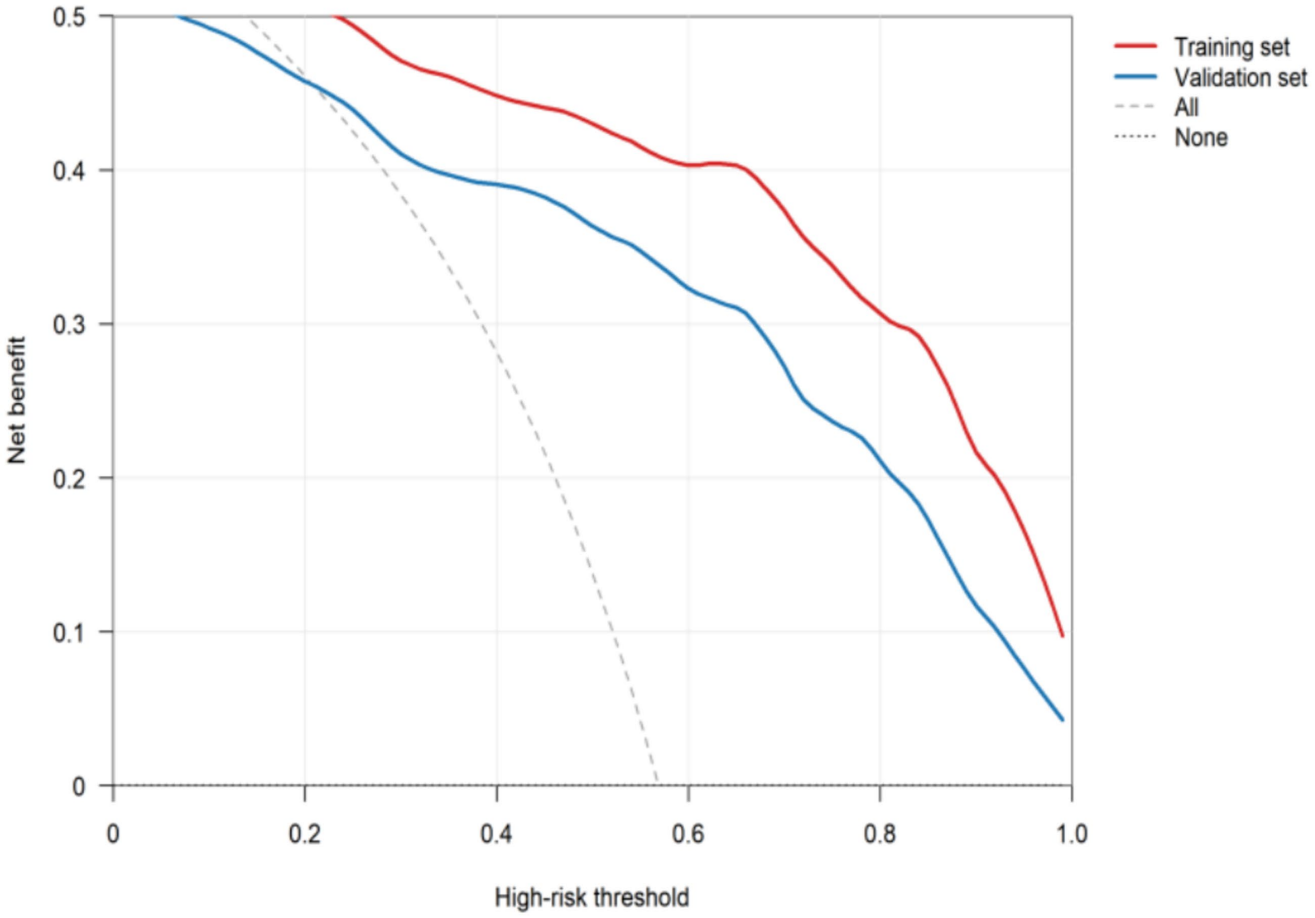
Decision curve analysis.

In the internal temporal validation cohort, the model had an AUC of 0.9086 (95% CI: 0.865–0.952), with a sensitivity of 85.5% and a specificity of 83.7% (Figure 5). Calibration analysis showed a calibration intercept of −0.1283, calibration slope of 0.8895, Brier score of 0.1220, and mean absolute error of 0.034. The Hosmer-Lemeshow goodness-of-fit test yielded chi-square = 4.613 and *p* = 0.798. These results indicated acceptable agreement between the estimated and observed probabilities in the temporal validation cohort; however, the calibration slope below 1.0 suggests slight overestimation of predicted risks. The calibration curves showed that the estimated and observed values were relatively close, indicating acceptable calibration (Figure 6). Decision curve analysis showed that the model provided greater net benefit than both the treat-all and treat-none strategies across threshold probabilities of 0.03–0.99 in the training cohort and 0.06–0.96 and 0.98–0.99 in the internal temporal validation cohort (Figure 7). This suggests that, across a broad range of clinically plausible decision thresholds, using the model to guide further risk assessment may provide more benefit than assuming that all or no patients have NSSI addiction-like features. Clinically, patients with an estimated probability below 0.30 may be considered low risk, those between 0.30 and 0.60 may be considered moderate risk, and those above 0.60 may be considered high risk; these cut-offs are exploratory, should be evaluated prospectively (for example, by examining calibration across risk deciles or other threshold-specific strata), and are not yet ready for routine clinical implementation.

## 4 Discussion

The LASSO-logistic model constructed in this study showed promising ability to stratify the cross-sectional likelihood of NSSI addiction-like features among adolescents and young adults with depression. The results indicated that age at first self-injury, gender, NSSI severity, borderline personality features, family functioning, and childhood trauma were significantly associated with NSSI addiction-like features. However, because predictors and outcome were measured at the same assessment point, the model should be interpreted as an association-based risk assessment model rather than as evidence that these variables causally or prospectively predict NSSI addiction-like features. The nomogram may help clinical healthcare workers identify patients who warrant more detailed assessment and targeted intervention, but further prospective testing and independent validation in other clinical settings are required.

This study included 511 adolescents and young adults with depression, of whom 284 (55.58%) met the operational criterion for NSSI addiction-like features. This proportion was lower than that reported in a previous study (69.2%) (Peng, 2023) but was comparable to another report (55.29%) (Ding et al., 2024). These differences may partly reflect differences in sample source and illness severity: the present sample and the study by Ding et al. (2024) were based mainly on outpatients, whereas the study by Peng (2023) focused mainly on inpatients. Because inpatients generally have more severe illness than outpatients, a higher prevalence of NSSI addiction-like features may be expected. In addition, social support, family functioning, and emotion regulation ability may vary across samples and may influence the occurrence of addictive features of NSSI (Burke et al., 2015; He et al., 2025; Lloyd-Richardson et al., 2007). Overall, the results of this study further support the view that NSSI is common and may show addiction-like features in adolescents and young adults with depression, suggesting that clinical interventions should emphasize early identification of NSSI and targeted psychological intervention to reduce the occurrence of NSSI addiction-like features and suicide risk in adolescents and young adults with depression.

The results of this study indicate that age at first self-injury is a protective factor against NSSI addiction-like features; that is, an older age at first self-injury is associated with a lower risk of addiction, which is consistent with previous findings (Zetterqvist, 2015; Nock, 2010). A possible explanation is that adolescence is a critical stage in the development of the brain reward circuitry and emotion regulation system, characterized by strong neural plasticity (Victor and Klonsky, 2014). When self-injurious behavior occurs in early adolescence, individuals may not yet have developed mature emotion regulation strategies and may obtain immediate emotional relief through pain-induced endorphin release during self-injury. This negative reinforcement effect may lead to behavioral dependence after repeated stimulation (Gao et al., 2025). In contrast, adolescents who first self-injure at an older age may have acquired richer peer support, recreational activities, and emotion regulation strategies. Thus, the substitute value of self-injury may be lower, and the risk of addiction may be relatively reduced (Brown and Sellbom, 2023). This finding suggests that future clinical practice should pay special attention to younger adolescents who engage in self-injury for the first time and should implement early interventions to interrupt the cycle of behavioral reinforcement.

Gender was also an important influencing factor for NSSI addiction-like features in adolescents and young adults with depression in this study. Logistic regression showed that males had a lower risk of NSSI addiction-like features than females, indicating that female adolescents and young adults with depression had a higher risk of NSSI addiction-like features. Previous systematic reviews and meta-analyses have shown that female gender is an important risk factor for adolescent NSSI, and most studies suggest that adolescent girls are more likely than boys to engage in NSSI (Wen et al., 2026). This may be related to greater emotional sensitivity, more prominent internalizing problems, stronger perceived interpersonal stress, and a heavier burden of depressive symptoms among pubertal girls. Relevant studies have indicated that adolescent girls are more likely to report internalizing symptoms, and that NSSI in girls is closely associated with depressive symptoms, interpersonal stress, and emotion regulation difficulties (Beauchaine et al., 2019; Burke et al., 2015). When facing negative emotions, female adolescents and young adults with depression may be more likely to direct distress toward themselves and to express emotions or relieve psychological stress through self-injury. NSSI is often regarded as a maladaptive way of coping with negative emotions and regulating emotional distress (He et al., 2025). Therefore, in the clinical management of adolescents and young adults with depression, particular attention should be paid to the concealed, recurrent, and addictive tendencies of NSSI among female patients, and emotion regulation training and crisis intervention should be implemented promptly.

NSSI severity usually reflects the frequency of self-injury, degree of tissue damage, diversity of methods, and difficulty controlling the behavior. Previous studies assessing NSSI severity have commonly used self-injury frequency, number of methods, injury consequences, and difficulty with behavioral control as important indicators (Lloyd-Richardson et al., 2007). Individuals with more severe NSSI may have developed a relatively stable pattern of self-injurious behavior. Their self-injury may no longer be an occasional stress response but may gradually develop into a recurrent, impulsive, or even dependent behavioral pattern. Studies have shown that individuals with repeated NSSI often exhibit stronger urges to self-injure, greater behavioral repetition, and addiction-like negative reinforcement features (Zetterqvist, 2015; Nock, 2010). Previous studies have also indicated that NSSI severity is closely associated with impaired psychological functioning, personality pathology, and trauma-related symptoms, and that depressive symptoms, borderline personality traits, childhood trauma, and emotion regulation difficulties are all associated with NSSI severity or the risk of recurrence (Victor et al., 2019; Gao et al., 2025).

The possible conceptual proximity between NSSI severity and NSSI addiction-like features deserves careful consideration. In this study, NSSI addiction-like features were defined using the seven-item OSI addiction subscale, whereas NSSI severity was calculated from OSI content reflecting behavioral frequency, methods, and difficulty controlling NSSI. The addiction subscale items themselves were not entered as predictors. Nevertheless, greater severity and poorer behavioral control are clinically related to addictive features such as persistence and loss of control; thus, the strong contribution of NSSI severity may partly reflect conceptual relatedness rather than an entirely independent risk domain. This issue could inflate model performance and should be regarded as a potential source of apparent over-optimism. For this reason, the model is presented as a cross-sectional risk stratification tool rather than as a causal or prospective prediction model, and future studies should evaluate whether comparable performance is retained after excluding severity items that are conceptually close to addiction-like features.

Borderline personality disorder features were a risk factor for NSSI addiction-like features in this study. These features are mainly characterized by emotional instability, impulsivity, unstable interpersonal relationships, intense fear of abandonment, and recurrent self-injury or suicide-related behaviors. The borderline pattern in ICD-11 also emphasizes core features including instability in interpersonal relationships, self-image, and affect, as well as marked impulsivity (Brown and Sellbom, 2023). Adolescents with depression who have prominent borderline personality traits may be more likely to use NSSI as an immediate emotion regulation strategy when facing mood fluctuations, interpersonal setbacks, or impaired self-evaluation. Previous studies have shown that borderline personality features can directly affect the risk of NSSI in adolescents and young adults with depression and can also indirectly affect this risk through emotion regulation ability (Chen et al., 2023). Borderline personality traits have been shown to play an important role in adolescent NSSI, particularly among adolescents and young adults with depression, in whom borderline personality traits are closely associated with negative life events and NSSI behaviors (Chen et al., 2024). Another study reported that borderline personality tendencies may increase NSSI intensity, whereas improving family functioning and subjective well-being may help reduce NSSI risk (Deng et al., 2023). Therefore, for adolescents and young adults with depression who show marked borderline personality features, interventions targeting emotion regulation, impulse control, interpersonal skills, and alternative behaviors to self-harm should be implemented as early as possible; evidence-based interventions such as dialectical behavior therapy may be considered when necessary.

Family functioning was a protective factor against NSSI addiction-like features in this study. The results showed that better family functioning was associated with a lower risk of NSSI addiction-like features in adolescents and young adults with depression. The family is an important source of emotional support, behavioral norms, and psychological security for adolescents. Support, communication, and supervision within the family environment are considered closely related to adolescent mental health and problem behaviors (Guo et al., 2026). Good family functioning can provide adolescents with stable emotional support, effective communication, and problem-solving resources, helping them adopt positive coping strategies when facing depressive mood, academic pressure, or interpersonal setbacks (Wu et al., 2026). In contrast, poor family functioning may involve insufficient parent–child communication, lack of emotional support, frequent family conflict, or caregiver neglect, all of which may weaken adolescents’ psychological resilience and make them more likely to use NSSI to relieve painful emotions (Victor et al., 2019). Previous studies have shown that family functioning is closely associated with adolescent NSSI, and depressive symptoms, emotional competence, and trauma-related symptoms may mediate the relationship between family functioning and NSSI (Wang Y. et al., 2022; Halstead et al., 2014). Therefore, interventions for NSSI addiction-like features in adolescents and young adults with depression should not be limited to individual therapy; family participation should also be emphasized, and improving parent–child communication, strengthening family support, and reducing family conflict may help lower the risk of NSSI addiction-like features.

Childhood trauma was also a risk factor for NSSI addiction-like features in adolescents and young adults with depression in this study. Childhood trauma may leave individuals in a long-term state of hypervigilance, low perceived safety, and negative self-cognition, increasing depression, anxiety, shame, emotion regulation difficulties, and self-punitive tendencies. Previous studies have indicated that childhood trauma is closely associated with trauma symptoms, emotional dysregulation, shame experiences, and NSSI risk (Andersson et al., 2022; Shi et al., 2024). Prior research has found that childhood trauma, especially emotional abuse and emotional neglect, is an important independent risk factor for NSSI in adolescents and young adults with depression (Shao et al., 2021). Other studies have suggested that childhood trauma may influence NSSI behaviors and depression severity in adolescents with major depressive disorder through maladaptive coping styles (Peng et al., 2024). For adolescents and young adults with depression who have experienced trauma, NSSI may not only serve as a way to relieve distress but may also be related to self-punishment, trauma re-experiencing, or emotional numbness. Studies have suggested that NSSI often functions to regulate negative emotions, punish the self, and cope with trauma-related distress (Tomás et al., 2016). Therefore, clinical healthcare professionals should adopt a trauma-informed care approach, avoid simplistic or punitive responses to self-injurious behavior, and help patients build a sense of safety, identify trauma-related emotions, and develop alternative emotion regulation strategies.

At present, the identification of NSSI addiction-like features in adolescents and young adults with depression mainly relies on clinical experience. Few studies by domestic scholars have reported risk prediction models for NSSI addiction-like features, and no suitable clinical auxiliary tools have been identified to help healthcare workers screen high-risk populations at an early stage. In this study, a risk assessment model was constructed using LASSO-logistic regression, and a nomogram was plotted based on the model. The AUC values in the training cohort and internal temporal validation cohort were 0.9366 and 0.9086, respectively, indicating good discriminatory ability in both datasets. The calibration curves showed that the estimated probabilities were close to the observed probabilities, and the Hosmer-Lemeshow goodness-of-fit tests were not statistically significant, indicating acceptable overall model fit. Bootstrap validation yielded an optimism-corrected AUC of 0.9310 for the final model and a more conservative optimism-corrected AUC of 0.9181 when the entire modeling procedure including LASSO selection was repeated. The Brier scores were 0.1000 in the training cohort and 0.1220 in the internal temporal validation cohort, and the validation calibration intercept and slope were −0.1283 and 0.8895, respectively. The calibration slope below 1.0 suggests slight overestimation of predicted risks in the internal temporal validation cohort, which reinforces the need for independent validation in other clinical settings before clinical implementation. VIF values ranged from 1.029 to 1.167, suggesting no apparent multicollinearity among the final predictors. Decision curve analysis showed clinical net benefit across threshold probabilities of 0.03–0.99 in the training cohort and 0.06–0.96 and 0.98–0.99 in the temporal validation cohort, supporting the potential clinical utility of the model for risk stratification. The exploratory low-, moderate-, and high-risk thresholds (<0.30, 0.30–0.60, and >0.60) should be prospectively evaluated, for example by examining calibration and observed event rates across risk deciles or other clinically relevant strata, and should not yet be treated as fixed clinical cut-offs.

## 5 Limitations

This study has several limitations. First, this was a cross-sectional study; therefore, temporal, causal, or prospective predictive relationships between age at first self-injury, family functioning, childhood trauma, borderline personality features, and NSSI addiction-like features cannot be established. Future prospective cohort studies are needed to verify whether these factors predict the onset, persistence, or escalation of NSSI addiction-like features. Second, although a temporal validation cohort was used, both cohorts came from the same institution and shared similar recruitment procedures. Therefore, the validation should be regarded as internal temporal validation rather than true independent validation in external cohorts, and independent validation in different regions and clinical settings is required. Third, the high AUC values suggest good discrimination but also raise the possibility of overfitting. Bootstrap-corrected estimates, calibration intercept and slope, Brier score, and VIF diagnostics were added to strengthen model evaluation; however, future studies should further evaluate model transportability. Fourth, NSSI addiction-like features were operationalized using the OSI addiction subscale and should not be interpreted as a formal DSM-5 or ICD-11 diagnosis. Fifth, the very small number of participants reporting a non-heterosexual orientation may reflect underreporting or limited measurement options, and conclusions related to sexual orientation should be avoided. Future studies should use more inclusive measures that assess sexual identity, attraction, and behavior separately to better capture LGBTQ+ representation in adolescent depression samples. Sixth, the inclusion of 20-year-old patients may limit generalizability to the strict WHO definition of adolescence. A separate age-restricted sensitivity analysis excluding participants older than 19 years was not performed in the current study; future studies should report discrimination and calibration metrics in a < 20-year subgroup to evaluate age-related generalizability. Seventh, the preliminary low-, moderate-, and high-risk thresholds derived from the decision-curve analysis are exploratory and require prospective testing, including evaluation of calibration and observed event rates across risk strata or deciles, before clinical adoption. Finally, the model mainly included clinical psychological and family-related variables. Future research may incorporate impulsivity, emotion regulation ability, sleep status, Internet use, peer relationships, biological indicators, and treatment response to improve model performance and clinical utility. In addition, because NSSI severity and NSSI addiction-like features are clinically related constructs measured using different OSI components, potential conceptual overlap and data leakage cannot be completely excluded. Future prospective studies should test alternative predictor sets that exclude variables closely related to the outcome definition.

## 6 Conclusion

The incidence of NSSI addiction-like features was relatively high among adolescents and young adults with depression in this single-center clinical sample. Age at first self-injury, gender, NSSI severity, borderline personality features, family functioning, and childhood trauma were important variables associated with NSSI addiction-like features. The LASSO-logistic model based on these factors showed promising discrimination, calibration, and clinical utility for cross-sectional risk stratification. Nevertheless, owing to the cross-sectional design and lack of independent validation in other clinical settings, the model should be applied cautiously and should be prospectively validated before being used as a routine clinical decision-making tool.

## Data Availability

The raw data supporting the conclusions of this article will be made available by the authors, without undue reservation.
