# Supplementary material for: Construction and internal temporal validation of a LASSO regression-based risk assessment model for non-suicidal self-injury addiction-like features in adolescents and young adults with depression
Source: Front Psychol. 2026 Jul 22;17:1895600. doi: 10.3389/fpsyg.2026.1895600 (PMC13437272; doi:10.3389/fpsyg.2026.1895600)
Supplement: Supplementary file 1 [file Data_Sheet_1.PDF]

Supplementary Materials

Supplementary Table S1. Missing values for candidate predictors, the outcome variable, and variables included in the multiple-imputation model.

Variable	Total N	Missing n	Missing %	Included in multiple-imputation model
NSSI addiction-like features	511	0	0.00	Yes
Age at first self-injury	511	0	0.00	Yes
Gender	511	0	0.00	Yes
Sexual orientation	511	0	0.00	Yes
Monthly household income	511	7	1.37	Yes
Parental educational level	511	5	0.98	Yes
Depression score	511	0	0.00	Yes
Anxiety score	511	0	0.00	Yes
NSSI severity score	511	0	0.00	Yes
Adverse life events score	511	4	0.78	Yes
Borderline personality feature score	511	6	1.17	Yes
Rumination score	511	3	0.59	Yes
Family functioning score	511	2	0.39	Yes
Loneliness score	511	3	0.59	Yes
Peer relationship score	511	2	0.39	Yes
Attitude toward school score	511	2	0.39	Yes
School bullying	511	0	0.00	Yes
Academic pressure score	511	2	0.39	Yes
Interpersonal relationship score	511	2	0.39	Yes
Social support score	511	3	0.59	Yes
Childhood trauma score	511	4	0.78	Yes

Note. All candidate predictors and the outcome variable were included in the multiple-imputation model. Missing values were not observed for the outcome variable. Missing proportions for candidate predictors ranged from 0.00% to 1.37%, and no variable exceeded the 20% threshold.

Supplementary Table S2. Measurement instruments, source references, scoring ranges, score directions, and internal consistency coefficients in the present sample.

Variable	Instrument	Principal source/reference	Items / scoring range	Score direction	Cronbach's alpha
NSSI addiction-like features	Chinese version of the Ottawa Self-Injury Inventory addiction subscale	Guérin-Marion et al., 2018; Luo et al., 2024	7 items; addiction-like features defined as scores >2 on more than three items	Higher scores indicate stronger addiction-like features of NSSI	0.852
NSSI severity	OSI items related to NSSI frequency, methods, and difficulty controlling the behavior	Ottawa Self-Injury Inventory; severity-related OSI items	Composite score calculated from severity-related OSI items	Higher scores indicate more severe NSSI	0.824
Depression	17-item Hamilton Depression Rating Scale (HAMD-17), Chinese version	Hamilton, 1960; Zheng et al., 1988	17 items; 0-52	Higher scores indicate more severe depressive symptoms	0.885
Anxiety	Hamilton Anxiety Rating Scale (HAMA-14), Chinese version	Hamilton, 1959	14 items; 0-56	Higher scores indicate more severe anxiety symptoms	0.845
Borderline personality features	Chinese version of the PDQ-4+ borderline personality disorder subscale	Hyler, 1994	Items/scoring range according to PDQ-4+ BPD subscale	Higher scores indicate more prominent borderline personality features	0.892
Family functioning	Chinese version of the Family APGAR Index	Smilkstein, 1978	5 items; 0-10	Higher scores indicate better family functioning	0.824
Social support	Chinese version of the Social Support Rating Scale (SSRS)	Xiao, 1994	10 items; total score commonly ranges from 12 to 66	Higher scores indicate greater social support	0.832
Rumination	Chinese version of the Ruminative Responses Scale (RRS)	Nolen-Hoeksema & Morrow, 1991	22 items; 22-88	Higher scores indicate greater rumination	0.786
Childhood trauma	Chinese version of the Childhood Trauma Questionnaire-Short Form (CTQ-SF)	Bernstein et al., 2003	28 items; 25-125	Higher scores indicate more severe childhood trauma	0.782
Adverse life events	Chinese version of the Adolescent Self-Rating Life Events Checklist (ASLEC)	Liu et al., 1997	27 items; scoring range according to ASLEC scoring rules	Higher scores indicate greater exposure to adverse life events	0.852
Loneliness	Validated Chinese loneliness scale	Validated Chinese scale used in the present study	Items/scoring range according to the scale used	Higher scores indicate greater loneliness	0.758
Peer relationships	Validated Chinese peer relationship scale	Validated Chinese scale used in the present study	Items/scoring range according to the scale used	Higher scores indicate better peer relationships unless otherwise specified	0.885
Attitude toward school	Validated Chinese school attitude scale	Validated Chinese scale used in the present study	Items/scoring range according to the scale used	Higher scores indicate a more positive attitude toward school	0.745
Academic pressure	Validated Chinese academic pressure scale	Validated Chinese scale used in the present study	Items/scoring range according to the scale used	Higher scores indicate greater academic pressure	0.882
Interpersonal relationships	Validated Chinese interpersonal relationship scale	Validated Chinese scale used in the present study	Items/scoring range according to the scale used	Higher scores indicate better interpersonal relationships unless otherwise specified	0.785
School bullying	Structured questionnaire item	Self-designed general information questionnaire	Yes/no	Presence of school bullying was coded as yes	Not applicable

Supplementary Table S3. Multicollinearity assessment using variance inflation factors (VIF).

Variable	R-squared	VIF	Tolerance
Age at first self-injury	0.080	1.087	0.920
Gender	0.061	1.065	0.939
NSSI severity	0.143	1.167	0.857
Borderline personality features	0.034	1.035	0.966
Family functioning	0.066	1.071	0.934
Childhood trauma	0.028	1.029	0.972

Note. All VIF values were below the conventional threshold of 5, indicating no apparent multicollinearity among the final predictors.
